# An Evaluation of the Hemoglobin–Albumin–Lymphocyte–Platelet (HALP) Score in Cushing’s Syndrome and Mild Autonomous Cortisol Secretion

**DOI:** 10.3390/jcm14228207

**Published:** 2025-11-19

**Authors:** Sevgül Fakı, Abbas Ali Tam, Belma Özlem Tural Balsak, Gülsüm Karaahmetli, Feride Pınar Altay, Didem Özdemir, Oya Topaloğlu, Reyhan Ersoy, Bekir Çakır

**Affiliations:** 1 Department of Endocrinology and Metabolism, Ankara Bilkent City Hospital, Ankara 06800, Turkey; belmabalsak@gmail.com (B.Ö.T.B.); gulsumgedik85@gmail.com (G.K.); fpaltay@gmail.com (F.P.A.); 2 Department of Endocrinology and Metabolism, Faculty of Medicine, Ankara Yildirim Beyazit University, Ankara 06800, Turkey; endoali@hotmail.com (A.A.T.); sendidem2002@yahoo.com (D.Ö.); oyasude@gmail.com (O.T.); reyhanersoy@hotmail.com (R.E.); drcakir@gmail.com (B.Ç.)

**Keywords:** HALP score, Cushing’s syndrome, mild autonomous cortisol secretion

## Abstract

**Background/Objectives:** Cushing’s syndrome (CS) is a rare endocrine disorder caused by chronic glucocorticoid excess. With the increasing recognition of mild autonomous cortisol secretion (MACS), clinical and biochemical differentiation between overt and mild forms has become more challenging. This study evaluated the clinical significance of the hemoglobin–albumin–lymphocyte–platelet (HALP) score in patients with Cushing’s disease (CD), adrenal Cushing’s syndrome (ACS), MACS, and nonfunctioning adrenal adenoma (NFA), focusing on its potential role in the preoperative evaluation and postoperative follow-up of hypercortisolism. **Methods:** We retrospectively analyzed 361 patients evaluated for cortisol excess between February 2019 and June 2025. Patients were categorized into four groups: CD, ACS, MACS, and NFA. Demographic, clinical, and hormonal parameters, as well as surgical outcomes, were recorded, and the HALP score was compared between the four groups. The diagnostic performance of the HALP score in differentiating overt Cushing’s syndrome (CD + ACS) from MACS/NFA was assessed using receiver operating characteristic (ROC) curve analysis. Postoperative changes in the HALP score were analyzed in surgically treated patients. **Results:** HALP scores were significantly lower in overt CS than in MACS and NFA. Using a threshold value of 40, the HALP score demonstrated 51.9% sensitivity and 90.4% specificity in differentiating CD/ACS from MACS/NFA. Among 68 operated patients, postoperative HALP data were available for 49 patients, for whom HALP scores significantly increased in both CD and ACS groups (*p* = 0.001 for each). **Conclusions:** The HALP score serves as a simple, cost-effective biomarker that reflects the combined hematologic and metabolic impact of cortisol excess. Significant postoperative improvement in the HALP score suggests its potential utility as a complementary tool in the preoperative assessment of hypercortisolism.

## 1. Introduction

Cushing’s syndrome (CS) is a rare endocrine disorder caused by chronic exposure to excessive glucocorticoids, either exogenous or endogenous [1]. After excluding exogenous glucocorticoid intake, CS is classified into ACTH-dependent and ACTH-independent forms, with approximately 80–85% of cases being ACTH-dependent, most commonly due to pituitary adenomas (Cushing’s disease—CD) [1,2]. In recent years, with the increasing recognition of mild autonomous cortisol secretion (MACS), the clinical, biochemical diagnosis, and therapeutic management of CS have become an even greater challenge for clinicians [3]. The clinical signs and symptoms of cortisol excess are highly variable, ranging from overt Cushingoid features such as in ectopic ACTH syndrome, where cortisol secretion is markedly elevated, to subtle or absent findings such as in patients with MACS [4,5,6].

When CS is suspected, first-line screening tests include the 1 mg overnight dexamethasone suppression test (DST), 24-h urinary free cortisol measurement (UFC), late-night salivary cortisol assay (SC), and midnight serum cortisol assessment. If the initial screening results suggest hypercortisolism, second-line investigations such as serum ACTH measurement, the CRH stimulation test, the high-dose (8 mg) DST, the desmopressin stimulation test, and bilateral inferior petrosal sinus sampling (IPSS) may be required to confirm the diagnosis and differentiate between ACTH-dependent and ACTH-independent forms [6].

Screening for CS is particularly recommended in adults who present with features unusual for their age (e.g., osteoporosis, hypertension), in those with multiple or progressive symptoms suggestive of hypercortisolism, and in patients with adrenal incidentalomas [6]. However, given the rising prevalence of obesity, diabetes, and hypertension in the general population, universal screening for CS is impractical and may result in false positive results and unnecessary further investigations. To minimize unnecessary testing and shorten diagnostic delays, alternative approaches have been explored, including clinical scoring systems, automated facial recognition, radiological or clinical assessment of skin thickness, quantification of facial plethora using near-infrared imaging, and, more recently, hematologic markers such as white blood cell-derived indices [7,8].

Cortisol excess exerts profound effects on immune regulation, hematopoiesis, and systemic metabolism. In general, glucocorticoids reduce peripheral blood lymphocyte counts [9], increase albumin catabolism [10], play a role in coagulation abnormalities, and stimulate erythropoiesis [11,12,13,14].

The hemoglobin–albumin–lymphocyte–platelet (HALP) score is a composite hematologic and nutritional index that has been validated as a prognostic biomarker in multiple malignancies, where lower HALP values consistently correlate with poorer outcomes, malnutrition, and systemic inflammation [15,16,17]. Beyond oncology, the HALP score has also been associated with cardiovascular and metabolic diseases, suggesting its potential as a global marker of inflammation and nutritional reserve [18,19,20].

Given the hematologic and metabolic alterations induced by cortisol excess, the HALP score may provide an integrative reflection of the magnitude and duration of glucocorticoid exposure by assessing its effects on hemoglobin (via erythropoiesis stimulation), albumin (via increased catabolism), lymphocytes (via apoptosis and redistribution), and platelets (via enhanced production), thereby serving as a composite marker of both endocrine activity (degree of hypercortisolism) and systemic burden (immunometabolic consequences) in patients with Cushing’s syndrome.

This study aimed to evaluate the HALP score in patients with Cushing’s syndrome and to assess its potential utility as a complementary tool in the preoperative assessment of hypercortisolism.

## 2. Materials and Methods

Between February 2019 and June 2025, patients diagnosed with CS, MACS, and NFA who were evaluated at the Endocrinology Department of Ankara City Hospital within a multidisciplinary council were retrospectively reviewed. Of the 476 patients initially evaluated, those with chronic kidney failure, hepatic failure, severe heart failure, active malignancy, pheochromocytoma, primary aldosteronism, pregnancy, autoimmune or rheumatologic disease, Factor V Leiden mutation or a history of thromboembolic events, incomplete diagnostic evaluation despite abnormal 1 mg DST results, and presentation of marked anemia (defined as hemoglobin < 10 g/dL), lymphopenia, thrombocytosis, or thrombocytopenia were excluded from the study. In total, 361 patients were included in the final analysis.

Demographic characteristics (age, sex, and body mass index [BMI]) and routine biochemical parameters were recorded, including morning fasting glucose (70–99 mg/dL), HbA1c (<5.7%), alanine aminotransferase (ALT) (<50 U/L), creatinine (0.5–1.1 mg/dL), albumin (32–48 g/L), potassium (3.5–5.5 mEq/L), hemoglobin (12–15.6 g/dL), lymphocytes (1.1–4.4 × 10^9^/L), and platelets (150–400 × 10^9^/L). Serum cortisol (5.2–22.4 µg/dL), ACTH (<46 pg/mL), dehydroepiandrosterone sulfate (DHEAS) (98.3–413.4 µg/dL), 1 mg DST (<1.8 µg/dL), UFC (<45 µg/24 h), midnight serum cortisol (normal < 7.5 µg/dL), and SC (<0.41 µg/dL) were also recorded. Complete blood count (CBC) analysis was performed using a Sysmex XN-Series automated hematology analyzer (Roche Diagnostics, Mannheim, Germany). Spectrophotometric measurements for fasting glucose, ALT, total cholesterol, and albumin levels were performed using the Siemens Atellica CH analyzer (Siemens Healthineers, Erlangen, Germany). Serum potassium (K) levels were measured using an indirect ion-selective electrode (ISE) method on the Siemens Atellica CH/CI analyzer (Siemens Healthineers, Erlangen, Germany). HbA1c was measured using high-performance liquid chromatography (HPLC) on the Tosoh G8 analyzer (Tosoh Bioscience, Tokyo, Japan).

Morning serum cortisol measurements were performed using the Siemens Atellica IM 1600 analyzer with immunoassay methodology (Siemens Healthineers, Erlangen, Germany). Twenty-four–hour urinary cortisol analysis was conducted using liquid chromatography–mass spectrometry (LC-MS/MS) with an Agilent LC-MS system (Agilent Technologies, Santa Clara, CA, USA). Night salivary cortisol was measured using the Roche Cobas e411 analyzer based on the electrochemiluminescence immunoassay (ECLIA) method (Roche Diagnostics, Mannheim, Germany).

Clinical features associated with CS—such as obesity, hirsutism, facial plethora, moon face, buffalo hump, purple striae, easy bruising, proximal muscle weakness, and centripetal obesity—were systematically evaluated. Furthermore, comorbid conditions including type 2 diabetes mellitus, hypertension, dyslipidemia, coronary artery disease, and osteoporosis were documented. In patients who underwent surgery, postoperative albumin, hemoglobin, lymphocyte, and platelet levels were also recorded. Patients with adrenal lesions showing a 1 mg DST cortisol level below 1.8 µg/dL (50 nmol/L) and no biochemical or clinical evidence of cortisol excess were defined as those with NFA. Cortisol hypersecretion was evaluated according to the diagnostic algorithm recommended by current guidelines and classified into three groups: CD, ACS, and MACS. Adrenal incidentaloma patients presenting with overt clinical signs and symptoms of hypercortisolism were classified as having ACS, whereas those without clinical manifestations were classified as having MACS. To differentiate CD from ectopic CS, an overnight high-dose DST and a CRH stimulation test were performed; when CRH was unavailable, a desmopressin stimulation test was used as a suitable alternative. Localization procedures were conducted as needed, with techniques such as IPSS utilized when required.

According to the results of the 1 mg DST, serum cortisol levels were classified as follows: 1.8–5 µg/dL as mild elevation, 5–10 µg/dL as moderate elevation, and >10 µg/dL as marked elevation.

According to 24-h urinary free cortisol (UFC) results, values were categorized as follows: ≤1× upper reference limit (normal), 1–3× (mildly elevated), and >3× (markedly elevated).

To determine the diagnostic performance of the HALP score in distinguishing patients with overt Cushing’s syndrome (CD and ACS) from those with NFA and MACS, receiver operating characteristic (ROC) curve analysis was performed.

The HALP score was calculated according to the following formula:

HALP = (Hemoglobin g/dL × Albumin g/L × Lymphocyte10^9^/L)/Platelet10^9^/L

## 3. Statistical Analysis of Demographic, Biochemical, and Hormonal Parameters

All statistical analyses were performed using IBM SPSS Statistics version 21.0 (Armonk, NY, USA: IBM Corp.). Continuous variables were tested for normality using the Shapiro–Wilk test and via visual inspection of histograms and Q–Q plots. Normally distributed variables (e.g., albumin, hemoglobin, lymphocyte count, and platelet count) were expressed as the mean ± standard deviation (SD) and compared across groups using Welch’s ANOVA, which provides robust results when variances are unequal. Post hoc pairwise comparisons for significant ANOVA findings were conducted using the Games–Howell test.

Variables not following a normal distribution were summarized as the median (minimum–maximum or interquartile range) and analyzed using non-parametric Kruskal–Wallis tests. When overall group differences were significant, Dunn–Bonferroni post hoc tests were applied for pairwise comparisons.

Categorical variables were compared using the Pearson Chi-square test or Linear-by-Linear Association where ordinal patterns existed. For paired (pre- vs. postoperative) HALP score comparisons within the same subgroup, the Wilcoxon Signed-Rank test was used due to non-normality and small sample sizes.

All tests were two-tailed, and a *p* value < 0.05 was considered statistically significant.

Compact ordering and directional arrows in the tables summarize the rank order of group medians or means in significant comparisons.

To evaluate whether the association between the HALP score and Cushing’s syndrome was confounded by sex distribution, we performed three complementary analyses: (1) multivariable logistic regression including both sex and HALP score as independent predictors of overt CS (CD/ACS vs. MACS/NFA); (2) sex-stratified logistic regression analyses conducted separately for males and females to assess the consistency of the HALP score–CS association across sexes; and (3) a comparison of HALP score between males and females within the control group (NFA) using independent samples *t*-test to determine whether baseline sex differences in HALP score exist in the absence of hypercortisolism.

To evaluate the diagnostic performance of the HALP score in distinguishing overt Cushing’s syndrome (CD and ACS) from NFA and MACS, receiver operating characteristic (ROC) curve analysis was performed.

The optimal cut-off value of the HALP score was determined using the Youden index (J = sensitivity + specificity − 1), which identifies the threshold maximizing overall diagnostic accuracy.

The following diagnostic performance parameters were reported: the area under the curve (AUC), 95% confidence interval (CI), sensitivity, specificity, positive predictive value (PPV), negative predictive value (NPV), accuracy, and likelihood ratios (LR^+^, LR^−^).

## 4. Results

This study included a total of 361 patients evaluated for cortisol excess. Based on hormonal and clinical findings, the cohort was divided into four groups: MACS, *n* = 30 (8.3%); ACS, *n* = 37 (10.2%); CD, *n* = 44 (12.2%); and NFA, *n* = 250 (69.3%). The median age was 56.5 years in the MACS group, 53.0 years in the ACS group, 52.0 years in the CD group, and 55.0 years (range 18–82) in the NFA group, with a borderline overall difference (Kruskal–Wallis *p* = 0.041). A statistically significant difference was observed regarding sex distribution, showing a clear female predominance across all subgroups: 21/30 (70.0%) in MACS, 35/37 (94.6%) in ACS, 39/44 (88.6%) in CD, and 153/250 (61.2%) in NFA (χ^2^ = 26.1, *p* < 0.001).

There were a total of 111 CS patients. Female predominance was also notable across subgroups of CS (*p* = 0.013) (Table 1). Significant intergroup differences were observed in BMI (*p* < 0.001), glucose (*p* = 0.033), ALT (*p* = 0.009), and potassium (*p* = 0.001). Overall, the CD and ACS groups showed higher BMI, glucose, ALT, and potassium levels compared with MACS, while lipid and HbA1c values were similar across groups (Table 1).

ACTH, morning serum cortisol, and DHEAS levels showed highly significant intergroup differences (*p* < 0.001 for each), with marked elevations in the CD group (Table 2). In the 1 mg DST, post-dexamethasone cortisol values were highest in the CD group (*p* < 0.001). Moreover, 24-h UFC levels displayed a similar gradient (CD > ACS = MACS; overall *p* = 0.014).

When stratified by the degree of elevation relative to the upper reference limit, 22.4% of all patients exhibited a marked (>3×) increase. The frequency of marked elevation was significantly higher in the CD group compared with both ACS and MACS groups, whereas no significant difference was observed between the ACS and MACS groups.

Late-night serum cortisol information was available in 59 subjects and showed a robust stepwise increase across groups (CD > ACS > MACS; *p* < 0.001), whereas SC values did not differ significantly (*p* = 0.462).

Obesity and centripetal obesity were significantly more frequent in the ACS group, whereas other clinical signs and symptoms—including plethora, moon face, buffalo hump, striae, and proximal muscle weakness—were more commonly observed in the CD group. When the total number of Cushingoid manifestations was considered, patients with CD demonstrated a significantly higher overall symptom burden, indicating a more overt Cushingoid appearance (*p* < 0.001; Table 3).

Comorbidity frequencies in patients with CS were as follows: type 2 diabetes mellitus (38.7%), hypertension (58.6%), dyslipidemia (31.5%), coronary artery disease (9.0%), and osteoporosis (19.8%). Although these conditions were common across the cohort, no statistically significant differences were observed between the CD, ACS, and MACS groups (all *p* > 0.05).

Overall, the total comorbidity burden did not differ significantly among groups (Kruskal–Wallis, *p* = 0.858) (Table 4).

Post hoc Bonferroni-adjusted analyses indicated higher female frequency and DST cortisol levels in CD and ACS compared with NFA; lower hemoglobin, lymphocyte counts, and HALP scores in CD; and higher albumin and creatinine in NFA (all *p* < 0.05) (Table 5). The HALP score differed significantly across groups (KW, *p* < 0.001). The score was lowest in CD (37.78 ± 22.45) and progressively higher in ACS (47.01 ± 24.78), MACS (55.13 ± 23.47), and NFA (58.36 ± 22.32). Pairwise comparisons of the HALP score in different groups confirmed that CD < ACS (*p* = 0.009), CD < MACS (*p* < 0.001), CD < NFA (*p* < 0.001), and ACS < NFA (*p* < 0.001).

Given the significant female predominance observed in the overt CS groups (88.6% in CD and 94.6% in ACS), we performed additional analyses to determine whether the observed association between HALP score and Cushing’s syndrome was independent of sex.

**Multivariable logistic regression analysis** including both sex and HALP score revealed that the HALP score remained a significant independent predictor of overt CS after controlling for sex (OR = 0.928, 95% CI: 0.907–0.950, *p* < 0.001). Similarly, female sex was independently associated with overt CS (OR = 5.71, 95% CI: [ekleyin], *p* < 0.001).

**Sex-stratified analyses** were performed to assess whether the effect of the HALP score differed between males and females. In males (*n* = 113), the HALP score remained a significant predictor of overt CS (OR = 0.914, 95% CI: 0.846–0.987, *p* = 0.022). In females (*n* = 248), the association was even stronger (OR = 0.930, 95% CI: 0.907–0.952, *p* < 0.001). The similarity in effect sizes (OR ≈ 0.91–0.93) across both sexes indicated no significant sex–HALP score interaction (Table 6).

**The comparison of HALP scores by sex in the control group (NFA, *n* = 280)** showed no significant difference between males (59.52 ± 17.23, *n* = 106) and females (58.01 ± 16.83, *n* = 174; t = 0.721, *p* = 0.471). This finding demonstrates that in the absence of hypercortisolism, HALP scores are comparable between sexes, confirming that the lower HALP values observed in overt CS are attributable to cortisol excess rather than sex distribution.

**Figure 1:**
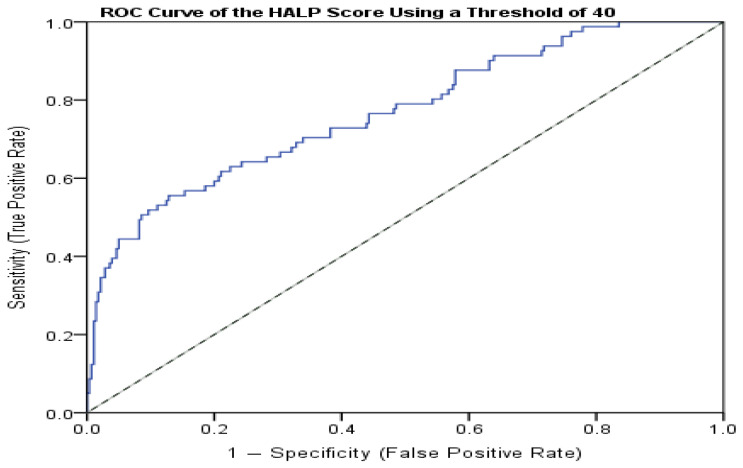
Receiver operating characteristic (ROC) curve of the HALP score for distinguishing overt Cushing’s syndrome (CD/ACS) from non-functioning adrenal adenoma (NFA) and mild autonomous cortisol secretion (MACS). The area under the curve (AUC) is 0.766 (95% CI: 0.705–0.828; *p* < 0.001). The optimal cut-off value of HALP ≤ 40 was determined using the Youden index.

Using ROC curve analysis, the optimal cut-off value of the HALP score to discriminate CD/ACS from NFA + MACS was determined to be 40, based on the maximum Youden index (Table 7, Figure 1).

Among the 111 patients included in this study, 68 (61.3%) underwent surgery, 34 (30.6%) were managed conservatively, and the surgical status was unknown in 9 patients (8.1%). When stratified by diagnostic subgroup, the proportions of operated patients were 50.0% in MACS, 56.8% in ACS, and 72.7% in CD.

Among the 34 nonoperated patients, the majority (44%) were considered to have subclinical Cushing’s syndrome, for which surgical intervention was not indicated and conservative follow-up was preferred. Other reasons included bilateral macroglandular hyperplasia (18%), refusal or inconclusive inferior petrosal sinus sampling (12%), diagnostic uncertainty regarding the source of hypercortisolism (9%), and patient refusal of surgery (9%). In a few cases, surgery was deferred due to comorbid conditions such as aortic aneurysm (3%) or ongoing preoperative evaluation (6%).

Among the 68 operated patients, biochemical remission was achieved in 52 (76.5%), persistent hypercortisolism was observed in 10 (14.7%), and postoperative dynamic test data were unavailable in 6 patients (8.8%).

Postoperative HALP scores were available for 49 patients; in the remaining 19 (27.9%), calculation was not possible due to incomplete data. Of these, 10 had persistent hypercortisolism, 6 lacked postoperative dynamic test results, and 3 were lost to follow-up, preventing postoperative HALP evaluation.

Postoperative HALP scores increased in all diagnostic groups, though the magnitude and statistical significance varied: In the MACS group, the median HALP score increased slightly from 53.1 to 57.1 (mean: 55.1 → 58.6), but the change was not statistically significant (*p* = 0.600). In the ACS group, the median HALP score rose from 43.7 to 48.6 (mean: 47.0 → 59.2), representing a clear postoperative increase that was statistically significant (*p* = 0.001). In the CD group, the median HALP score increased markedly from 37.8 to 49.4 (mean: 38.4 → 53.9), showing the strongest and significant postoperative improvement (*p* = 0.001) (Table 8).

## 5. Discussion

Measuring the complete blood count (CBC) is a routine test in clinical practice and is commonly performed in patients with suspected cortisol excess. In this study, we evaluated the HALP score in the context of endogenous cortisol excess. The HALP score was significantly lower in patients with CD and ACS compared with MACS and NFA. Given the high specificity (90.4%) and strong positive likelihood ratio (LR+ 5.41) at HALP ≤40, the HALP score might serve as a rule-in test for endogenous cortisol excess (CD/ACS). Although ACTH, serum cortisol, DHEAS, 1 mg DST, UFC, and late-night serum cortisol levels were all markedly higher in CD compared with ACS and MACS (CD > ACS ≈ MACS), late-night serum cortisol levels were notably lower in MACS than CD and ACS. Only 5.1% (2/39) of MACS patients had 1 mg DST cortisol levels exceeding 10 µg/dL and markedly elevated values of UFC were also rare among MACS patients, observed in only 2 of 22 cases (9%). SC levels were higher in the CD group than in the ACS and MACS groups; however, these differences did not reach statistical significance. This may be due to the small sample size, the sensitivity of the method used, and patients’ full cooperation with the test.

In the literature, there are no head-to-head comparisons assessing the performance of diagnostic tests. According to the ESE-ENSAT 2023 Guidelines, in patients diagnosed with MACS, a second clinical evaluation, measurement of ACTH, and, in those at risk of false positive 1 mg DST results, assessment of DHEA-S levels are recommended as supportive parameters. However, the guidelines emphasize that these parameters have limited accuracy in predicting existing comorbidities. Similarly, in our study, although these parameters were found to be higher in the CD group, no significant difference was observed in the prevalence of comorbidities between groups. All patients with MACS should be screened for cortisol-related comorbidities that are potentially attributed to cortisol (e.g., hypertension and type 2 diabetes mellitus) to ensure that these are appropriately treated. In patients with MACS who also have relevant comorbidities, surgical treatment should be considered as part of an individualized approach [3].

In recent years, easily accessible prediction tools and scoring systems have been proposed for a wide variety of diseases. These scores show promise for early detection and prediction of outcomes without the need for extra testing. The PLR (Platelet-to-Lymphocyte Ratio), NLR (Neutrophil-to-Lymphocyte Ratio), MLR (Monocyte-to-Lymphocyte Ratio), and SII (Systemic Immune-Inflammation Index) scores are all derived from simple CBC analyses. Additionally, the HALP score is calculated using CBC parameters together with a serum albumin measurement.

Although initially proposed as a prognostic factor in malignancies [21], the HALP score has since been evaluated for the discrimination of malignant and benign diseases [22] and as a prognostic marker in several conditions (appendicitis, stroke, coronary artery disease, dyslipidemia, and complications of diabetes) [23,24,25,26]. Similar to these conditions, inflammation is also increased in endogenous cortisol excess, which switches off multiple inflammatory genes, in addition to activating many anti-inflammatory genes [27].

In our cohort, hemoglobin, lymphocyte count, and serum albumin levels were significantly lower in patients with CD and ACS compared with those with MACS and NFA, while platelet counts were relatively higher in the hypercortisolemic groups. These findings are consistent with the known hematologic effects of chronic cortisol excess.

Long-term exposure to glucocorticoids (GCs) results in decreased expression of several proinflammatory cytokines, increased production of immunosuppressive proteins, and the induction of proinflammatory lymphocyte apoptosis in peripheral blood [28]. In addition, GCs have the potential to inhibit intercellular adhesion of lymphocytes to endothelial binding sites by suppressing the expression of lymphocyte adhesion molecules [29]. The potential mechanism by which endogenous GC exposure may reduce peripheral blood lymphocyte counts has been investigated at the molecular level. In a study analyzing peripheral blood-derived CD34^+^-enriched hematopoietic stem and progenitor cells (HSCDs), researchers examined how GCs regulate genes involved in cell survival and apoptosis. Specifically, they evaluated the mRNA expression levels of the pro-apoptotic BAX gene and the anti-apoptotic BCL-xL and BCL-2 genes. In peripheral blood hematopoietic progenitor cells obtained from patients with adrenal dysfunction, a marked increase in the mRNA level of the pro-apoptotic BAX gene was identified. In cells derived from patients with ACTH-independent CS, BAX gene expression increased by approximately 35% compared with healthy controls. Even more strikingly, in ACTH-dependent CS patients, BAX gene expression was elevated by nearly 60%. These findings indicate that chronic endogenous GC excess may enhance the expression of apoptosis-related genes in hematopoietic progenitor cells, thereby contributing to a reduction in lymphocyte populations [9]. Similarly, in our study, lymphocyte counts were significantly reduced in patients with ACTH-dependent CS.

The relationship between CS and hemoglobin levels has been investigated in several studies. In a previous study, elevated erythrocyte counts were reported in patients with overt CS [13], and in two separate reports, polycythemia was described as one of the earliest manifestations of the disease [30,31]. In one study investigating sex-specific discrepancies, it was observed that females with CD had higher hematocrit (HCT), RBC, and hemoglobin (Hb) levels compared with control subjects, whereas males with CS had lower HCT, RBC, and Hb levels than their control counterparts. This difference was suggested to be related to the gonadal status in men, which may exert a greater influence on hematologic function than hypercortisolism itself [32]. Conversely, an Italian study evaluating a cohort of 80 patients with active CD reported anemia and reduced RBC counts in men, while women did not show any significant hematologic alterations [33]. In our study, a clear female predominance was observed in the cortisol excess groups, whereas the NFA group consisted of 61% females, and the mean age was lower in patients with CD compared with NFA. Therefore, the significantly lower hemoglobin levels observed in our cohort may be explained by menstrual cycle-related factors in women. The significant female predominance in the overt CS groups (88.6% in CD and 94.6% in ACS) raised the question of whether the observed HALP differences might be confounded by sex rather than reflecting true cortisol-related pathophysiology. To address this concern, we performed multivariable regression, sex-stratified analyses and baseline sex comparisons in the control group. These analyses collectively demonstrated that the HALP score remained a significant predictor of overt CS after adjusting for sex, showed consistent effects in both males and females, and critically did not differ between sexes in the absence of hypercortisolism (NFA group: *p* = 0.471). These findings confirm that the lower HALP scores in CD and ACS reflect genuine cortisol-induced hematologic and metabolic alterations rather than sex-related confounding, supporting the validity of the HALP score as a biomarker in this setting.

Most previous studies in patients with CS have focused on coagulation abnormalities and the hypercoagulable state, whereas investigations specifically addressing platelet counts are relatively scarce. Sato et al. reported significant alterations in several biochemical parameters in patients with CS, including increased platelet counts and changes in γ-glutamyltranspeptidase, choline esterase, creatine phosphokinase, and lactate dehydrogenase levels. These findings were attributed to the systemic metabolic and inflammatory effects of chronic hypercortisolism [1]. Consistent with these observations, our study also revealed significantly higher platelet counts in patients with ACTH-independent CS and CD compared with NFA (*p* < 0.001; ACS ≈ CD > NFA), supporting the link between hypercortisolism and enhanced platelet production.

In a study using I^131^-labeled albumin, albumin turnover was evaluated to assess the dynamic characteristics of albumin metabolism in CS. Patients with active disease showed a markedly shortened biological half-life, reflecting accelerated albumin degradation. A similar pattern was observed in exogenous corticosteroid-induced CS, where higher steroid doses produced a more pronounced effect. The total albumin pool was reduced, particularly in patients with shorter half-lives and more severe clinical manifestations. These findings indicate that excessive glucocorticoid exposure enhances albumin catabolism, partially compensated by increased hepatic synthesis, leading to a net protein imbalance manifested clinically as osteoporosis, muscle wasting, and skin thinning [10]. Similarly, in our study, serum albumin levels were significantly lower in both ACTH-independent CS and CD groups compared with the NFA group.

Given that dynamic endocrine tests—such as the 1 mg DST, UFC measurement, late-night serum measurement, or SC assay—are often time-consuming, expensive, and may yield discordant results, the HALP score offers a simple, cost-effective, and easily accessible surrogate marker derived from routine laboratory parameters. The HALP score could reflect both disease activity and systemic impact in patients with endogenous cortisol excess. Nevertheless, this hypothesis requires confirmation through larger, prospective studies before the HALP score can be considered a reliable adjunct in the diagnostic or monitoring algorithms for CS and MACS.

Following curative surgery, HALP values significantly increased in both CD and ACS groups, reflecting postoperative recovery of hematologic and nutritional parameters. The observed rise in hemoglobin, lymphocyte, and albumin levels together with normalization of platelet counts supports the hypothesis that the HALP score can serve not only as a static indicator of cortisol-related burden but also as a dynamic marker of recovery after biochemical remission. These findings are consistent with the reversibility of glucocorticoid-induced immunometabolic alterations described in previous studies.

This study has several strengths. It is, to our knowledge, the first to evaluate the HALP score in patients with endogenous cortisol excess, including both preoperative and postoperative assessments, thereby demonstrating its potential as a dynamic marker. It also comprises a relatively large cohort of patients with CS, a rare condition, allowing for meaningful comparison between the CD, ACS, and MACS groups. Moreover, all patients were evaluated within a multidisciplinary endocrine council involving endocrinologists and surgeons, ensuring diagnostic accuracy.

However, this study has certain limitations. Its retrospective, single-center design may introduce selection bias and limit generalizability. In addition, patient classification—particularly between MACS and ACS—was based on council documentation and clinical interpretation, leaving a minimal possibility of misclassification. Data on nutritional status, menopausal state, and smoking habits were not available, which may represent additional unmeasured confounders. Larger, prospective multicenter studies are warranted to validate these findings and further define the role of the HALP score in monitoring treatment outcomes. Despite the female predominance in our cohort, which is consistent with the known epidemiology of Cushing’s syndrome, we confirmed through comprehensive sex-adjusted analyses that the association between the HALP score and overt CS was independent of sex distribution.

## 6. Conclusions

Although biochemical cortisol burden was higher in ACS and CD compared with MACS, the HALP score decreased in parallel with cortisol excess, reflecting the hematologic and metabolic impact of hypercortisolism. Despite comparable rates of comorbidities across all three groups, the HALP score distinguished patients with overt hypercortisolism from those with mild autonomous secretion. These findings suggest that, in addition to conventional hormonal tests, a low HALP score may provide supportive evidence when considering surgical intervention in patients with suspected cortisol excess. Integrating the HALP score into the routine biochemical evaluation may help identify patients with clinically significant cortisol excess earlier and guide individualized management.

## Figures and Tables

**Table 1:** Demographic and laboratory parameters in subgroups of Cushing’s syndrome.

Variable	CD (*n* = 44, 39.6%)	ACS (*n* = 37, 33.3%)	MACS (*n* = 30, 27.0%)	*p*	Compact Ordering
Age (years) *	52 (22–70)	53 (22–74)	56.5 (36–74)	0.148	≈
**Female, *n* (%)**	**39 (88.6%)**	**35 (94.6%)**	**21 (70.0%)**	**0.013** (χ^2^)	**ACS ≈ CD > MACS**
BMI (kg/m^2^) *	33.0 (23.8–49.6)	34.2 (23.4–48.4)	28.5 (21.5–39.5)	**<0.001**	**CD ≈ ACS > MACS**
Glucose (mg/dL) *	95.0 (66–273)	96.0 (73–264)	86.5 (70–147)	**0.033**	**ACS > MACS ≈ CD**
Creatinine (mg/dL) *	0.660 (0.40–0.95)	0.700 (0.40–1.30)	0.675 (0.40–1.00)	0.723	≈
ALT (U/L) *	25 (12–156)	22 (12–93)	20 (8–45)	**0.009**	**CD > ACS ≈ MACS**
**TKOL (mg/dL)**	201.9 ± 46.1	197.3 ± 39.9	192.4 ± 43.6	0.648	≈
**LDL (mg/dL)**	118.1 ± 40.8	118.2 ± 31.3	116.2 ± 38.1	0.971	≈
HDL (mg/dL) *	49.0 (24–78)	47.0 (26–93)	44.5 (30–72)	0.776	≈
TG (mg/dL) *	149.5 (69–486)	129.0 (66–322)	146.0 (61–311)	0.610	≈
A1C (%) *	6.0 (5.1–10.8)	6.0 (5.1–9.4)	5.9 (5.2–7.7)	0.368	≈
K (mmol/L) *	4.50 (2.1–5.34)	4.40 (3.6–5.1)	4.10 (2.8–4.7)	**0.001**	**CD ≈ ACS > MACS**

CD—pituitary Cushing’s disease; ACS—adrenal Cushing; MACS—mild autonomous cortisol secretion. Values are expressed as the median (range) * or mean ± SD (unmarked; one-way ANOVA). ns—not significant.

**Table 2:** Basal hormonal profile and screening tests in subgroups of Cushing’s syndrome.

Test (Unit)	CD	ACS	MACS	*p* (Kruskal–Wallis)	Compact Ordering
Plasma ACTH (pg/mL) * (*n* = 111)	53.0 (12.2–189.0) (*n* = 44)	6.2 (0.0–14.6) (*n* = 37)	6.2 (5.0–14.9) (*n* = 30)	**<0.001**	**CD > ACS ≈ MACS**
Morning serum cortisol (µg/dL) * (*n* = 111)	22.45 (7.8–63.0) (*n* = 44)	14.6 (6.0–30.9) (*n* = 37)	12.85 (6.5–26.5) (*n* = 30)	**<0.001**	**CD > ACS ≈ MACS**
DHEAS (µg/dL) * (*n* = 105)	174.5 (3–621) (*n* = 40)	22.0 (0–590) (*n* = 35)	42.0 (10–214) (*n* = 30)	**<0.001**	**CD > ACS ≈ MACS**
DST 1 mg cortisol (µg/dL) * (*n* = 111)	13.5 (2–73) (*n* = 44)	3.9 (1.9–30.9) (*n* = 37)	3.53 (1.9–28.0) (*n* = 30)	**<0.001**	**CD > ACS ≈ MACS**
1.8–5 µg/dL (mild elevation)	13 (29.5%)	24 (64.9%)	21 (70%)		
5–10 µg/dL (moderate)	4 (9.1%)	3 (8.1%)	7 (23%)		
>10 µg/dL (marked)	27 (61.4%)	10 (27.0%)	2 (6.7%)	*p* < 0.001	**CD > ACS > MACS**
24-h urinary free cortisol (µg/24 h) * (*n* = 104)	86.5 (17–2279) (*n* = 40)	56.0 (4.4–565) (*n* = 34)	52.5 (12.4–442) (*n* = 30)	**0.014**	**CD > ACS ≈ MACS**
≤1× (normal)	11 (27.5%)	12 (35.5%)	15 (50%)		
1–3× (mild high)	13 (32.5%)	15 (44.1%)	10 (33%)		
>3× (marked high)	15 (37.5%)	5 (14.7%)	2 (6.7%)	*p* = 0.019	**CD > ACS ≈ MACS**
Late-night serum cortisol (µg/dL) * (*n* = 59)	17.1 (7.0–57.0) (*n* = 32)	10.7 (2.4–49.1) (*n* = 14)	5.6 (2.8–24.4) (*n* = 13)	**<0.001**	**CD > ACS > MACS**
Late-night salivary cortisol (µg/dL) * (*n* = 65)	0.66 (0.00–8.40) (*n* = 29)	0.48 (0.02–4.17) (*n* = 23)	0.39 (0.02–1.58) (*n* = 20)	**0.462**	**CD ≈ ACS ≈ MACS**

CD—hypophyseal Cushing; ACS—adrenal Cushing; MACS—mild autonomous cortisol secretion. Values are expressed as the median (range) * or mean ± SD (unmarked; one-way ANOVA).

**Table 3:** Comparison of clinical characteristics and post hoc analysis in subgroups of Cushing’s syndrome.

Feature	CD (*n* = 44)	ACS (*n* = 37)	MACS (*n* = 30)	*p* Value	Post Hoc
Obesity	26 (59.1%)	26 (70.3%)	9 (30.0%)	<0.001	ACS ≫ CD ≫ MACS
Hirsutism *	8 (18.2%)	4 (10.8%)	0 (0%)	0.033	CD > ACS > MACS
Plethora	13 (29.5%)	7 (18.9%)	0 (0%)	0.005	CD ≫ ACS ≫ MACS
Moon face	20 (45.5%)	18 (48.6%)	0 (0%)	<0.001	CD ≈ ACS ≫ MACS
Buffalo hump	20 (45.5%)	16 (43.2%)	0 (0%)	<0.001	CD ≈ ACS ≫ MACS
Striae *	10 (22.7%)	4 (10.8%)	0 (0%)	0.011	CD > ACS > MACS
Easy bruising *	7 (15.9%)	4 (10.8%)	0 (0%)	0.051	Trend: CD > ACS > MACS
Proximal muscle weakness	12 (27.3%)	2 (5.4%)	1 (3.3%)	0.003	CD ≫ ACS ≈ MACS
Centripetal obesity	13 (29.5%)	17 (45.9%)	0 (0%)	<0.001	ACS ≫ CD ≫ MACS
**Total symptom count (median [IQR])**	5 (3–6)	3 (2–4)	1 (0–2)	<0.001	CD > ACS > MACS

* Variables with low expected cell counts (<20), for which Fisher’s Exact Test was applied. *p* values were calculated using Fisher’s Exact Test (due to expected cell counts <5 in ≥20% of cells); all other comparisons used Pearson’s Chi-square test. Continuous variables (total symptom count) compared by Kruskal–Wallis test. ≫—highly significant difference (*p* < 0.001); >—significant difference (*p* < 0.05); ≈—no significant difference (*p* ≥ 0.05).

**Table 4:** Distribution of comorbidities and total comorbidity burden by group.

Comorbidity	CD (*n* = 44)	ACS (*n* = 37)	MACS (*n* = 30)	*p*	Significant Pattern
Type 2 Diabetes Mellitus	19 (43.2%)	15 (40.5%)	9 (30.0%)	0.501	NS
Hypertension	25 (56.8%)	22 (59.5%)	18 (60.0%)	0.955	NS
Dyslipidemia	13 (29.5%)	12 (32.4%)	10 (33.3%)	0.933	NS
Coronary Artery Disease	5 (11.4%)	1 (2.7%)	4 (13.3%)	0.249	NS (trend CD ≈ MACS > ACS)
**Osteoporosis**	**12 (27.3%)**	**6 (16.2%)**	**4 (13.3%)**	**0.268**	**NS (trend CD > ACS > MACS)**
**Total Comorbidity Count (Mean ± SD)**	**1.68 ± 1.44**	**1.51 ± 1.22**	**1.50 ± 1.38**	**0.858**	**NS (Kruskal–Wallis *p* > 0.05)**

CD—pituitary Cushing’s disease; ACS—adrenal Cushing; MACS—mild autonomous cortisol secretion. NS—not significant.

**Table 5:** Demographic and laboratory characteristics across study groups (MACS, ACS, CD, and NFA).

Variable	MACS	ACS	CD	NFA	*p*	Compact Ordering (sig. Only)
Sex (female %)	21 (70.0%)	35 (94.6%)	39 (88.6%)	153 (61.2%)	**<0.001**	ACS ≈ CD ≈ MACS > NFA
Age (years) *	56.5 (36–74)	53.0 (22–74)	52.0 (22–70)	55.0 (18–82)	**0.041**	NFA > CD
Glucose (mg/dL) *	86.5 (70–147)	96.0 (73–264)	95.0 (66–273)	92.0 (67–381)	**0.042**	ACS ≈ CD ≈ NFA > MACS
Creatinine (mg/dL) *	0.675 (0.40–1.00)	0.700 (0.40–1.30)	0.660 (0.40–0.95)	0.775 (0.46–1.34)	**<0.001**	NFA > ACS ≈ MACS ≈ CD
Albumin (g/L)	44.70 ± 2.98	44.08 ± 3.10	42.90 ± 4.34	46.03 ± 2.40	**<0.001**	NFA > CD and NFA > ACS
ALT (U/L) *	20 (8–45)	22 (12–93)	25 (12–156)	23 (6–237)	**0.027**	CD > ACS ≈ NFA ≈ MACS
Hemoglobin (g/dL)	13.91 ± 1.50	13.38 ± 1.12	13.22 ± 1.18	14.19 ± 1.23	**<0.001**	CD, ACS < MACS, NFA
Lymphocytes (×10^9^/L)	2.28 ± 0.70	2.25 ± 0.57	1.90 ± 0.55	2.25 ± 0.64	**0.007**	CD < ACS ≈ MACS ≈ NFA
Platelets (×10^9^/L)	263.3 ± 79.6	290.5 ± 58.0	295.1 ± 87.3	253.3 ± 53.3	**<0.001**	ACS ≈ CD > NFA
DST 1 mg (µg/dL) *	3.53 (1.90–28.00)	3.90 (1.90–30.95)	13.50 (2.00–73.00)	1.07 (0.40–1.78)	**<0.001**	CD > ACS ≈ MACS > NFA
HALP score *	55.13 (23.47)	47.01 (24.78)	37.78 (22.45)	58.36 (22.32)	**<0.001**	CD < ACS < MACS ≈ NFA

Values are expressed as the median (range) * or mean ± SD (unmarked). Glucose values ACS > MACS, *p* = 0.008; NFA > MACS, *p* = 0.043. Creatinine values showed a stepwise increase, being highest in NFA (KW, *p* < 0.001; NFA > CD, *p* < 0.001; NFA > ACS, *p* = 0.001; NFA > MACS, *p* = 0.004). Albumin levels were significantly higher in NFA (Welch F(3,66.2) = 11.87, *p* < 0.001; NFA > CD, *p* < 0.001; NFA > ACS, *p* = 0.004). ALT was significantly high in CD (KW, *p* = 0.027; CD > ACS, *p* = 0.035; CD > MACS, *p* = 0.004; CD > NFA, *p* = 0.022). Hemoglobin levels were lowest in CD and ACS (Welch F(3,71.4) = 11.66, *p* < 0.001; NFA > CD, *p* < 0.001; NFA > ACS, *p* = 0.001). Lymphocyte counts were also reduced in CD (Welch F(3,357) = 4.06, *p* = 0.007; CD < ACS, *p* = 0.02; CD < MACS, *p* = 0.01; CD < NFA, *p* = 0.01). Platelet counts were higher in CD and ACS compared with NFA (Welch F(3,66.6) = 6.98, *p* < 0.001; CD > NFA, *p* = 0.018; ACS > NFA, *p* = 0.003). Finally, 1 mg DST cortisol levels were highest in CD and lowest in NFA (KW *p* < 0.001; CD > ACS, *p* = 0.003; CD ≫ MACS, *p* < 0.001; CD ≫ NFA, *p* < 0.001; MACS ≫ NFA, *p* < 0.001; ACS ≫ NFA, *p* < 0.001). Data are expressed as the mean ± SD or median (range), as appropriate. *p* values represent comparisons among the four groups using Welch ANOVA for normally distributed variables and the Kruskal–Wallis test for non-normal data; categorical variables were analyzed by the χ^2^ test.

**Table 6:** Sex-stratified analysis of HALP score and multivariable regression.

Analysis	Group	*n*	OR (95% CI)	*p* Value
**Multivariable regression**	HALP score	361	0.928 (0.907–0.950)	<0.001
(Both sex and HALP score included)	Female sex	361	5.71	<0.001
**Sex-stratified**	Males only	113	0.914 (0.846–0.987)	0.022
	Females only	248	0.930 (0.907–0.952)	<0.001
**Control group (NFA)**	Males	106	59.52 ± 17.23	0.471 ^a^
HALP score comparison	Females	174	58.01 ± 16.83	

^a^ Independent samples *t*-test comparing males vs. females.

**Table 7:** Distribution of HALP score (≤40 vs. >40) by overt Cushing’s status: CD/ACS versus NFA + MACS.

	HALP Score ≤ 40	HALP Score > 40	Total
**CD/ACS**	42	39	81
**NFA + MACS**	27	253	280
**Total**	69	292	361

Using a HALP threshold of 40 (positive—HALP ≤ 40), the discrimination was found to be good (AUC 0.766, 95% CI 0.705–0.828; *p* < 0.001). At this cut-off, the sensitivity was 51.9%, the specificity was 90.4%, the PPV was 60.9%, the NPV was 86.6%, the accuracy was 81.7%, the LR+ was 5.41, and the LR− was 0.53.

**Table 8:** Comparison of preoperative and postoperative HALP scores by diagnostic group (Wilcoxon Signed-Rank Test).

Group	*n*	Preop Median (IQR)	Postop Median (IQR)	*p* (2-Tailed)
**MACS**	13	53.1 (23.5)	57.1 (28.4)	0.600
**ACS**	18	43.7 (24.8)	48.6 (34.4)	**0.001**
**CD**	18	37.8 (22.5)	49.4 (22.1)	**0.001**

## Data Availability

The datasets generated and analyzed in the present study are available upon reasonable request to the corresponding author.
